# A Rare Case of Latent Tuberculosis Reactivation Secondary to a COVID-19 Infection

**DOI:** 10.3390/idr14030048

**Published:** 2022-06-12

**Authors:** Ana-Alicia Leonso, Kyle Brown, Raquel Prol, Saumya Rawat, Arjun Khunger, Romina Bromberg

**Affiliations:** 1 Department of Graduate Medical Education, Memorial Hospital West, Pembroke Pines, FL 33028, USA; rprol@mhs.net (R.P.); srawat@mhs.net (S.R.); akhunger@mhs.net (A.K.); 2 Department of Pharmacy, Memorial Hospital West, Pembroke Pines, FL 33028, USA; 3 Department of Infectious Diseases, Memorial Hospital West, Pembroke Pines, FL 33028, USA; robromberg@mhs.net

**Keywords:** COVID-19, latent tuberculosis, *Mycobacterium tuberculosis*, SARS-CoV-2

## Abstract

Coronavirus disease 2019 (COVID-19) and tuberculosis (TB) are currently the two leading causes of death among infectious diseases. As we progress towards a “new normal”, more information is required regarding post-COVID-19 syndromes. We present a case of latent tuberculosis reactivation 3 months after a successful inpatient treatment of COVID-19. A 74-year-old female from the Philippines presented with a new left mid-lung infiltrate with worsening shortness of breath and lethargy for one week prior to admission. The clinical course of the patient deteriorated despite broad-spectrum antibiotics, diuretics, and high-dose steroid therapy requiring intubation and mechanical ventilation. Her sputum culture yielded the microbiological diagnosis of TB. Anti-tubercular medications were started and the patient had a favorable clinical outcome. Our case demonstrates that immunosuppression secondary to COVID-19 and its treatments may promote the development of an active TB infection from a latent infection. It is important to be aware of this potential increase in risk during and after a COVID-19 treatment. This is especially important in high-risk populations to ensure an early diagnosis and prompt management as well as to reduce transmission.

## 1. Introduction

With nearly 180 million cases, the novel coronavirus disease 2019 (COVID-19) has significantly strained healthcare systems worldwide. Approximately 15–30% of patients can develop acute respiratory distress syndrome, requiring prolonged mechanical ventilation and lung recruitment strategies to overcome profound hypoxemia [1]. Currently, immunomodulators have shown the most promise in reducing the morbidity and mortality of COVID-19 [2,3,4,5]. However, with the increased use of these medications, there is a heightened concern for immunosuppression and superinfections. In addition, COVID-19 has been shown to cause lymphopenia and impair cytokine circulation during the hyperinflammatory phase of the illness, which may increase the risks of concomitant infections [6,7]. In the past, superinfections in the acute setting were linked to increased ICU admissions, hospital length of stay, and mortality. Furthermore, this level of immunosuppression puts patients at an increased risk of bacterial, fungal, viral, and opportunistic infections.

Tuberculosis (TB) remains the number one infectious cause of death worldwide despite global efforts to eradicate the disease. Approximately 25% of the global population has a latent tuberculosis infection (LTBI) with an estimated 5–15% lifetime activation risk [8]. As countries proceed towards a “new normal”, there may be an increased incidence of TB secondary to active or recovered COVID-19 infections, whether or not immunosuppressant medications are used. Additionally, given that patient access to screening and treatment of TB may be affected by strategies to mitigate the spread of COVID-19, there could be delays in the diagnosis of TB leading to an increased number of cases. Therefore, the World Health Organization (WHO) has recommended simultaneous testing for TB and COVID-19 when indicated as part of their strategy to improve treatment outcomes and reduce the spread of TB [8]. Screening for LTBI prior to or after a treatment for COVID-19 is not currently recommended in the guidelines for COVID-19 management. This paper describes a case of active TB presenting shortly after a successful treatment of COVID-19.

## 2. Case Presentation

**Figure 1:**
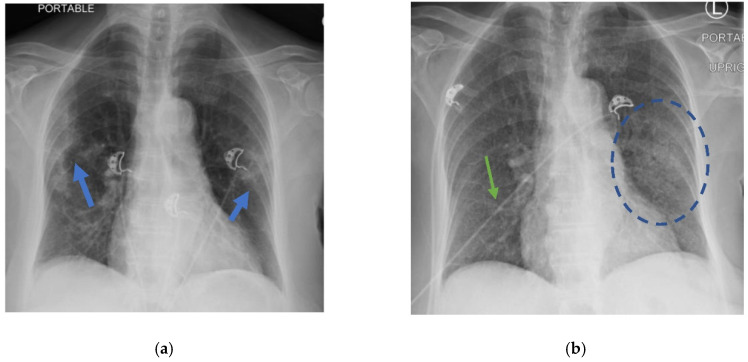
(**a**) Chest X-ray with multifocal ground glass infiltrates (blue arrows) during active COVID-19 infection; (**b**) chest X-ray 3 months after with resolution of prior patchy bilateral infiltrates with prominent interstitial markings (green arrow) unchanged from prior chest X-ray and new left mid-lung zone infiltrate (blue circle).

**Figure 2:**
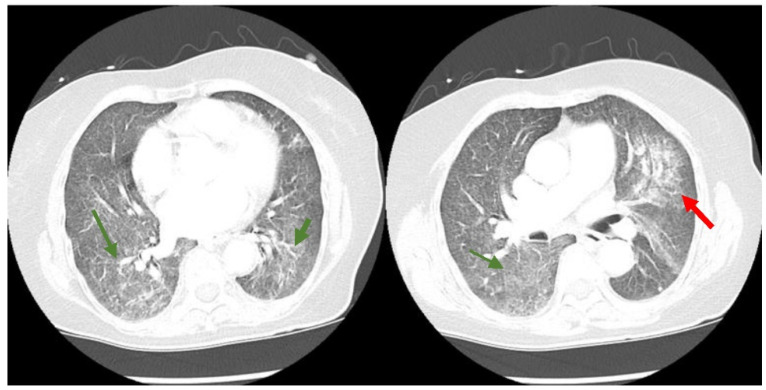
CT chest scan with diffuse ground glass opacities throughout both lungs more extensive than present on prior imaging (green arrows) with a superimposed patchy airspace consolidation in the perihilar region of the left upper lung lobe (red arrow).

**Figure 3:**
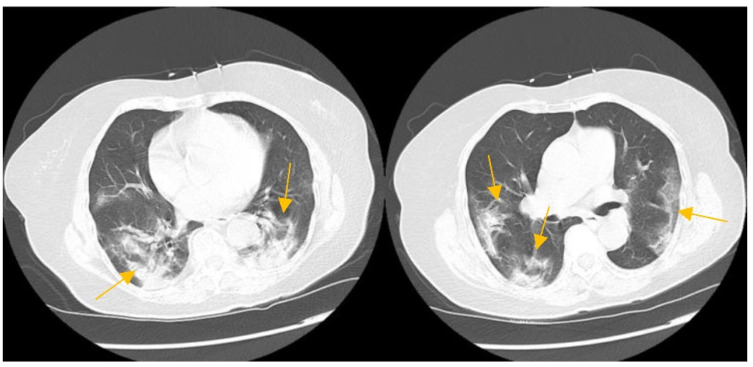
CT chest scan during active COVID-19 infection showing moderate multifocal areas of consolidation (yellow arrows).

A 74-year-old Filipino female with a medical history significant for hypertension, hyperlipidemia, and non-insulin-dependent type II diabetes presented to the emergency department (ED) of a South Florida hospital with a one-week history of increased generalized weakness and worsening shortness of breath. Three months prior, the patient was treated in the hospital for COVID-19 with intravenous dexamethasone and remdesivir, and was discharged home with oxygen. She fully recovered to her baseline and no longer required supplemental oxygen two months before the above onset of symptoms. On arrival to the ED, she was lethargic, but not in severe distress. She had an oral temperature of 37.5 °C, a heart rate of 97 beats/min, blood pressure of 133/78 mmHg, a respiratory rate of 20 breaths/min, and oxygen saturation of 91% on 3 L nasal cannula supplemental oxygen. She had decreased breath sounds bilaterally and rales at the bases. Her heart sounds were distant without any murmurs and she had no peripheral edema. The initial laboratory results are available in Table 1. Her chest X-ray showed a left mid-lung infiltrate that was new compared with the X-ray three months prior (Figure 1). The computed tomography (CT) chest scan with IV contrast showed no evidence of a pulmonary embolism. However, it revealed significant bilateral infiltrates (Figure 2) that had progressed from her previous CT chest scan three months prior (Figure 3). Her echocardiogram revealed an ejection fraction of 39% and no other structural abnormalities.

Throughout the hospitalization, her hypoxemia worsened for which she required BiPAP and subsequently developed paroxysmal atrial fibrillation with a rapid ventricular response. The acute hypoxemic respiratory failure of the patient was thought to be multifactorial and was empirically treated with diuretics, antibiotics, and steroids for a suspected pulmonary edema vs. pneumonia vs. nitrofurantoin-induced pneumonitis as the patient received nitrofurantoin for a UTI one week before arrival to the ED. She was also treated with therapeutic enoxaparin for a non-ST elevation MI although it was likely due to demand ischemia in the setting of severe pulmonary disease. She remained febrile despite optimal treatment, prompting a further workup including a lumbar puncture 15 days after admission; however, the CSF findings were unrevealing for an infectious etiology. The patient had a worsening clinical course requiring intubation and mechanical ventilation. An induced sputum culture sample obtained after intubation showed a positive AFB smear and positive PCR for a *Mycobacterium tuberculosis* (MTB) complex. Subsequent induced sputum cultures were also positive for MTB, confirming the diagnosis of TB.

Initially, the treatment regimen was rifamycin, isoniazid, pyrazinamide, and ethambutol (RIPE therapy) for MTB. However, the patient developed transaminitis within a few days so she started second-line therapy (linezolid, isoniazid with vitamin B6, and levofloxacin). The patient was also placed on pulse-dose steroids for presumed immune reconstitution inflammatory syndrome (IRIS) and *Pneumocystis jirovecii* prophylaxis with atovaquone. In order to improve efficacy, rifabutin was added, but ultimately the regimen was changed to ethambutol, rifabutin, and levofloxacin due to ongoing hepatotoxicity. Eventually, the respiratory status of the patient improved so she underwent a tracheostomy placement and subsequently improved enough to have the tracheostomy closed. Finally, the patient was discharged to a specialized TB inpatient center to complete six months of MTB treatment with rifabutin, levofloxacin, and ethambutol.

## 3. Discussion

The above case illustrates the likely risk of a LTBI reactivation in a patient who had recently recovered from COVID-19 treated with steroids and remdesivir. Possible causes were the immunosuppressive sequelae of COVID-19, the effects of the treatment, or a combination of both. In any case, it is essential to consider the risk of a TB infection when treating COVID-19 patients, especially those who have emigrated from TB-endemic areas. For example, the patient presented in this case was an immigrant from the Philippines without other risk factors or known recent exposures. Therefore, the most likely conclusion was that it was a reactivation of LTBI triggered by COVID-19 rather than acute TB.

Although TB is less common in the United States (US) than in the rest of the world, it is not rare, especially in areas with large immigrant populations. In 2018, the Centers for Disease Control and Prevention (CDC) reported 9029 new cases of TB in the US with two-thirds of cases in non-US-born people (a rate 14 times greater than US-born people), and 591 cases in the state of Florida [9]. Unfortunately, although it is required to report cases of active TB to the CDC, it is not required for cases of LTBI. Therefore, there are only incidence/prevalence estimates provided by the CDC via the National Health and Nutrition Examination Survey (NHANES), which includes a medical examination and TB screening tests; these data are extrapolated to the civilian population. In 2015, the estimated national prevalence of LTBI in the US was 13.2 million [10].

Recently, it has been shown that COVID-19 infections affect the innate immune system and T cells, which are essential to prevent LTBI reactivation. For example, studies have shown that SARS-CoV-2 can cause T cell dysfunction and cytokine storms, leading to immunosuppression. Patients with COVID-19 have decreased numbers of lymphocytes, specifically CD4 and CD8 T cells; there is evidence that these cells undergo “exhaustion”, becoming dysfunctional/ineffective, and express an increased level of immune-inhibitory factors on their surface. These patients also tend to have increased levels of cytokines such as TNF-α, IL-6, and IL-10 [6,11]. One case series showed that 46.4% of patients with COVID-19 developed a secondary infection with respiratory and bloodstream infections being the most common source; this risk was higher in patients receiving corticosteroids, but was also present in patients who did not receive corticosteroids. However, this case series had a high number of severe and critically ill patients, so it may not be representative of all COVID-19 infections [6]. Previous studies in mice have shown that a depletion of CD4 T cells is associated with the reactivation of LTBI. CD4 T cells are critical to the cytotoxic function of CD8 T cells via cytokines, and a depletion of CD8 T cells has also been associated with the reactivation of LTBI [12]. Multiple viruses—including other coronaviruses such as SARS-CoV and MERS-CoV as well as viruses such as HIV, influenza, and measles—have been shown to increase the risk of a reactivation of LTBI [13,14,15]. Other case series in TB-endemic areas such as China and Italy have increasingly documented COVID-19 and active TB co-infections. The majority of the current studies focus on co-infections or an increased risk of COVID-19 in patients with TB and vice versa, but little is documented on LTBI reactivation after recovery. In a study looking at patients with SARS-CoV, their cell counts took up to three months to normalize for CD8 T cell counts and up to one year for CD4 T cells [16]. Therefore, it is possible that an infection with SARS-CoV-2 alone could put patients with LTBI at an increased risk of reactivation. There are two other separate case reports of patients that developed an active TB infection several weeks after recovery from COVID-19 and these patients likely had LTBI (both were from endemic areas and one had a known exposure via a household contact two years prior; neither patient had previously been tested or treated for LTBI) [17,18].

Additionally, the treatment for patients with COVID-19 who require supplemental oxygen is steroids, which also have immunosuppressive effects. The Infectious Disease Society of America (IDSA) guidelines for COVID-19 treatment recommend corticosteroids for a critical illness or severe non-critical illness as well as tocilizumab, which directly blocks the IL-6 receptor, for severe disease with signs of systemic inflammation [19]. Corticosteroids mainly cause immunosuppression via effects on the function of phagocytes and temporary reductions in lymphocytes, which are necessary for preventing LTBI from reactivating via granuloma formation and other activities [20]. There are limited data regarding whether tocilizumab increases the risk of an active TB infection, but corticosteroids have been shown to increase the risk of new TB infections and the reactivation of LTBI, usually with prolonged and high-dose courses. If a patient is going to be on a prolonged course of high-dose corticosteroids and has an increased risk of LTBI, it is recommended that they be screened with an interferon-gamma release assay or tuberculin skin testing [21]. The same principle may need to be considered for patients treated for COVID-19.

## 4. Conclusions

In short, we present a case of TB in the US of a patient from the Philippines who recovered from a COVID-19 infection treated with corticosteroids and remdesivir and subsequently developed TB three months after recovery. Given the history and patient risk factors, this was likely a reactivation of LTBI rather than a new acute TB infection. There are currently no guidelines regarding whether screening for TB should be considered in patients with COVID-19, whether they receive treatment with steroids or tocilizumab. However, it seems that, given the increasing number of case reports regarding a co-infection with TB and the possible reactivation of LTBI, it may be prudent to begin screening high-risk patients with COVID-19, especially severe cases that require treatment. There are increasing data in the literature regarding co-infections of COVID-19 and TB, but a paucity of data regarding the possible reactivation of LTBI long after treatment and recovery from COVID-19; this will need further investigation.

## Figures and Tables

**Table 1:** Initial laboratory findings and results.

Laboratory Test	Results	Reference Values
Blood Urea Nitrogen	19 mg/dL	7–18 mg/dL
Creatinine	1.02 mg/dL	0.51–0.95 mg/dL
Sodium	131 mmol/L	136–145 mmol/L
Potassium	3.3 mmol/L	3.5–5.1 mmol/L
Chloride	99 mmol/L	98–107 mmol/L
Bicarbonate	21 mmol/L	21–32 mmol/L
White Blood Cell Count	8.5 × 10^3^/uL	3.5–10 × 10^3^/uL
Lactic Acid	6.5 mmol/L	0.4–2.0 mmol/L
Procalcitonin	21.88 ng/mL	<0.05 ng/mL
D-Dimer	1.7 mg/L	<0.49 mg/L
Pro-BNP	13,240 pg/mL	<352 pg/mL
Respiratory Pathogen Panel	Negative	Negative
Urine Legionella Antigen Test	Negative	Negative
SARS-CoV-2	Negative	Negative
Arterial Blood Gas *		
pH	7.42	7.35–7.45
pCO_2_	27 mmHg	32–45 mmHg
paO_2_	283 mmHg	75–85 mmHg
Troponin		
1st	<0.015 ng/mL	≤0.045 ng/mL
2nd	3 ng/mL	
3rd	2.48 ng/mL	

* Taken on BiPAP with 100% FiO_2._

## Data Availability

Not applicable.
